# Unlocking the Potential of Substrate Quality for the Enhanced Antibacterial Activity of Black Soldier Fly against Pathogens

**DOI:** 10.1021/acsomega.3c09741

**Published:** 2024-02-12

**Authors:** Mach P. Achuoth, Cynthia M. Mudalungu, Brian O. Ochieng, Hosea O. Mokaya, Shadrack Kibet, Vinesh J. Maharaj, Sevgan Subramanian, Segenet Kelemu, Chrysantus M. Tanga

**Affiliations:** † International Centre of Insects Physiology and Ecology, P.O. Box 30772-00100 Nairobi, Kenya; ‡ Biodiscovery Center, Department of Chemistry, Faculty of Natural and Agricultural Sciences, University of Pretoria, Private Bag X20, Hatfield 0028, South Africa; § Department of Chemistry, College of Science and Technology, Dr John Garang Memorial University of Science and Technology-Bor, P.O. Box 436 Juba, South Sudan; ∥ School of Chemistry and Material Science, The Technical University of Kenya (TUK), P.O. Box 52428-00100 Nairobi, Kenya

## Abstract

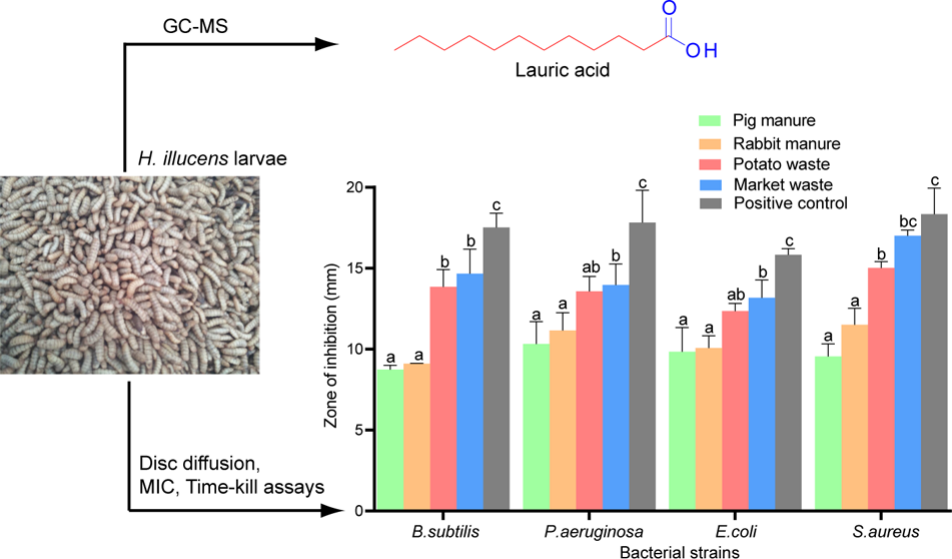

Globally, antibiotics are facing fierce resistance from multidrug-resistant bacterial strains. There is an urgent need for eco-friendly alternatives. Though insects are important targets for antimicrobial peptides, it has received limited research attention. This study investigated the impact of waste substrates on the production of antibacterial agents in black soldier fly (*Hermetia illucens* L.) larvae (HIL) and their implications in the suppression of pathogens [*Bacillus subtilis* (ATCC 6051), *Staphylococcus aureus* (ATCC 25923), *Pseudomonas aeruginosa* (ATCC 27853), and *Escherichia coli* (ATCC 25922)]. The 20% acetic acid (AcOH) extract from market waste had the highest antibacterial activity with an inhibition zone of 17.00 mm, followed by potato waste (15.02 mm) against *S. aureus*. Hexane extract from HIL raised on market waste also showed a significant inhibitory zone (13.06 mm) against *B. subtilis*. .Minimum inhibitory concentration (MIC) values recorded were 25 mg/mL against all test pathogens. The fastest time-kill of 20% AcOH extract was 4 h against*B. subtilis*, *E. coli*, ,and*P. aeruginosa*. Lauric acid was also identified as the dominant component of the various hexane extracts with concentrations of 602.76 and 318.17 μg/g in HIL reared on potato and market waste, respectively. Energy from the market waste substrate correlated significantly (*r* = 0.97) with antibacterial activities. This study highlights the key role of substrate quality and extraction methods for enhancing the production of antibacterial agents in HIL, thus providing new insights into the development of potential drugs to overcome the alarming concerns of antimicrobial resistance.

## Introduction

Antimicrobial resistance (AMR) is a serious global affliction in the 21st century affecting human beings, animals, and the environment at large.^1,2^ The overuse of antibiotics and their widespread availability over the counter are some of the contributing factors to this menace.^3,4^ According to projections, illnesses linked to AMR will claim approximately 10 million lives yearly by 2050.^5^ The *ESKAPE* pathogens that include *Enterococcus faecium*, *S. aureus*, *Klebsiella pneumoniae*, *Acinetobacter baumannii*, *P. aeruginosa*, *Enterobacter* spp., and *E. coli* have been implicated in this burden, contributing to an annual mortality rise of 2.1%.^6,7^ The number of established antimicrobial agents is diminishing at an alarming rate, attributed to the limited mode of action and target.^8,9^ Notwithstanding the efforts by the scientific community to continue with the innovation of antibiotics, bacteria have fought back by developing various mechanisms of resistance, thus inflicting a worldwide catastrophe.^10^ The mode of action of antimicrobial agents developing resistance by pathogenic bacteria includes: the breakdown of membranes, targeting of intracellular components, and interference with bacterial metabolism in which tested target compounds align themselves to prompt the death of bacteria.^11,12^ To counteract this scenario, there is a need to intensify the search for potent antimicrobial agents from the understudied niches to diversify therapeutic choices.^13^ This call encompasses the discovery of novel antimicrobial agents to curb the increasing global problem of bacterial, fungal, and viral resistance against drugs.^14,15^ In recent years, much attention has been devoted to the structural three-dimensional classification of antimicrobial peptides (AMPs) present in insects without their isolation.^13^ Insects are seen as potential sources of antimicrobial agents with promising action against the problems caused by antimicrobial resistance.^16−18^

Indeed, insects represent the largest class of living organisms in the animal kingdom, accounting for more than 50% of all described species.^19^ This naturally available niche could be tapped as an alternative source of bioactive metabolites. Already, cecropin A, first isolated from *Hyalophora cecropia* was found to disrupt the biofilm of *E. coli*.^20^ Similarly, one metabolite offered for the treatment of ailments by insects is the bioactive agent melittin, a major component of venom from *Apis millifera*, which showed anti-inflammatory properties.^21^

One of the insects that are visualized to possess these important natural bioactive metabolites is the black soldier fly, *H. illucens* (L.) (Diptera: Stratiomyidae). This insect has a propensity to thrive in decomposing waste environments, harboring a wide community of microorganisms.^22^ It has an innate immune system that produces potent antimicrobial agents to protect itself from pathogen invasions.^23^ In lieu of this, hexanedioic acid was previously isolated from *H. illucens* larval powder and exhibited antibacterial activity.^24^ Purified extracts from the hemolymph of *H. illucens* larvae vaccinated with *Lactobacillus casei* displayed antimicrobial activity against *K. pneumoniae*.^25^ Furthermore, it was currently reported that extracts containing sterols derived from *H. illucens* larvae (HIL) possess antibacterial activity against a panel of bacteria including the Gram-positive *B. subtilis*.^26^ Moreover, Choi et al.^25^ found that methanol extracts of *H. illucens* reared on unsegregated wastes exhibited inhibitory activity against Gram-negative bacteria. Their study did not however investigate the effects of segregating the rearing substrates on the antibacterial activity of the extracts, a factor that could potentially influence the expression of antibacterial metabolites in *H. illucens*. Further, there is a dearth of information on the effect of different rearing substrates and extraction solvents on the antibacterial activities of HIL extracts. This study therefore sought to investigate the effects of rearing substrates on the antibacterial activities of HIL extracted using different solvents and explore the correlation these activities have with the proximate composition of the rearing substrates.

## Materials and Methods

### Rearing of HIL

Twenty batches of freshly laid*H. illucens* eggs, each veiled in 5 cm long plastic wrinkled pipes, were collected from the black soldier fly colony at the International Centre of Insect Physiology and Ecology (*icipe*), Nairobi, Kenya. They were then dipped in plastic trays measuring 57 L × 37 W × 10 H cm. Six trays of each substrate were prepared by weighing 3.5 kg of fermented pig, rabbit manure, and potato and market wastes and poured into a separate plastic tray. The larvae were reared in a temperature-controlled room (temperature: 29 ± 2 °C; relative humidity: 65 ± 5%). The eggs were allowed to be hatched in 3–4 days, and the larvae were fed by adding 2.0 kg of each substrate into their respective trays every 3 days. The larvae were harvested at the fifth instar, washed with 70% ethanol, and rinsed with sterilized water. The cleaned larvae were dried in an oven (KAPD-195D; CNT Co., Ltd., Gwangju, Korea Republic) at 50 °C for 18 h, ground with a blender (500 W Trio Mixer Grinder; Preethi, Chennai, India), and stored until further use.

### Defatting of HIL Powder and Preparation of Extracts

HIL powder from larvae reared on different substrates was defatted by adopting the previous procedure with some modifications.^27^ Approximately 200 g of HIL from each substrate was soaked in 1 L of hexane for 24 h at room temperature. This mixture was filtered through Whatman filter paper and then concentrated in vacuo at 40 °C to obtain HIL hexane extracts that were subsequently kept at −20 °C for further use. The resultant sludge was dried in a fume hood for 12 h. The dry biomass was divided into two, 100 g each, and subsequently extracted with 500 mL of 20% acetic acid (AcOH) and 80% methanol (MeOH) for 24 h. The mixtures were filtered and then concentrated *in vacuo* to obtain the 20% AcOH and 80% MeOH extracts.

### Bacterial Strains Used for Biological Assays

Four bacterial pathogens, two Gram-positive bacteria, *B. subtilis* (*ATCC* 6051) and *S. aureus* (*ATCC* 25923), and two Gram-negative bacteria, *P. aeruginosa* (*ATCC* 27853) and *E. coli* (*ATCC* 25922), were used to test the antibacterial activities of HIL extracts. These test microorganisms were obtained from the *icipe* laboratory. Fresh bacterial colonies were cultured from the mother colonies on Mueller Hinton agar (CM0337B, Thermo Fisher Scientific, MA) in 90 mm Petri dishes.

### Determination of the Antibacterial Activities of Extracts

Using disc diffusion assays, the antibacterial activities of the HIL extracts obtained were evaluated. The bacteria were cultured overnight at 37 °C and standardized to an optical density (OD) of 0.09–1.03 at 630 nm. A suspension of 100 μL containing each 1.0 × 10^6^ CFU/mL bacterial strain was applied to Mueller Hinton Agar (MHA) Petri dishes. Eight sterile paper discs (6 mm) were then placed on the inoculated discs. Accurately, 20 μL of the prepared concentration (2 mg/disc) of each extract was measured using a pipet and applied on top of the paper discs. About 20 μL of 1.0 mg/mL chloramphenicol and 5% dimethyl sulfoxide (DMSO) were applied as positive and negative controls, respectively. The Petri dishes were incubated at 37 °C for 24 h, after which the zones of inhibitions were measured across the paper discs and recorded in millimeters. The tests were all repeated in triplicates.

### Determination of Minimum Inhibitory Concentration (MIC)

The minimum inhibitory doses were evaluated using the broth microdilution assay.^28^ The extracts from 20% AcOH with a known concentration of 100 mg/mL from HIL fed with plant-based substrates (market and potato wastes), and animal-derived substrates (pig and rabbit manures) were serially diluted up to 1.56 mg/mL using Mueller Hinton broth (MHB) media. About 80 μL (40 μL of MHB + 40 μL of extracts) of the aliquot was then transferred into each of the 96 well plates, and 10 μL of test bacterial pathogens (1.0 × 10^6^ CFU/mL) were added. The plates were then incubated in a rotary shaker at 37 °C for 24 h. Turbidity was compared with that of the positive control (broth medium, test bacterial strain, and 1.0 mg/mL chloramphenicol) and a negative control (test bacteria and broth medium). The MIC value was recorded as the lowest concentration of HIL extracts at which no observable growth in the tubes was seen. The experiments were done in three replicates.

### Determination of Minimum Bactericidal Concentration (MBC)

Minimal bactericidal concentration (MBC) is the minimum concentration of an antimicrobial agent that can stop the growth of the tested organism on an agar plate. This was determined by subculturing broth dilutions from MIC assays, where 10 μL was pipetted from clear to turbid wells and streaked on the MHA plate. Incubation was done at 37 °C for 24 h. The lowest HIL extract concentration showing no observable bacterial growth colonies on an MHA plate was recorded as MBC. The tests were done in triplicate.

### Time-Kill Analysis

A time-kill assay was performed on 20% AcOH HIL extracts against four test microorganisms according to the previously described method with slight modifications.^29^ In summary, the bacterial culture suspension was adjusted to approximately 1.0 × 10^6^ CFU/mL. The HIL extracts were diluted with MHB medium containing the inoculum to obtain final concentrations of 0 × MIC (0 mg/mL) for control and 1 × MIC (25 mg/mL) for HIL extracts from pig manure, rabbit manure, potato waste, and market waste. The cultures reached a final volume of 1 mL that contained 500 μL of MHB and the same quantity for extract. All samples were incubated at 37 °C with a shaking speed of 180 rpm in a rotary shaker. A time interval of bacterial growth and death was programmed from 0 to 12 h. At each 4 h interval, 100 μL of the aliquots were transferred to a microcentrifuge plate and diluted to 10^1^, 10^–2^, and 10^–4^ in 1% phosphate-buffered saline (PBS). About 100 μL of the resultant aliquot was taken and spread on the MHA plate, and the number of colonies formed on the plates after incubation at 37 °C for 24 h was counted and calculated. The graphs of log_10_ CFU/mL over time were plotted.

### Determination of the Fatty Acid Content of Extracts

Fatty acids present in HIL fed with four substrates and extracted using hexane were determined and measured as fatty acid methyl esters (FAMEs) using a modified version of the method.^30^ In brief, 1 mL of a sodium methoxide solution (15 mg/mL) was added to 300 mg of each sample. The mixture was then vortexed for 1 min, ultrasonicated for 10 min, and incubated in a water bath at 70 °C for 1 h. After that, 100 μL of deionized water was added to the mixture to quench it, followed by another 1 min of vortexing. To extract the resulting FAMEs, 1 mL of gas chromatography (GC)-grade hexane (Sigma-Aldrich, St. Louis, MO) was added to the mixture, which was then centrifuged at 14,000 rpm for 20 min. The surfactant was subsequently dried using anhydrous sodium sulfate and filtered. The resultant methylated fatty acids were analyzed by gas chromatography (GC) on a 7890A gas chromatography instrument (Agilent Technologies, Inc. Santa Clara, CA) coupled with a 5975 C mass selective detector (Agilent Technologies, Inc. Santa Clara, CA). The GC analysis was conducted at the following conditions: the inlet temperature was set at 270 °C, the transfer line temperature at 280 °C, and the column oven temperature was programmed to increase from 35 to 285 °C, with the initial temperature maintained for 5 min, followed by a ramp of 10 °C/min to 280 °C for 10 min. The final temperature was increased at a rate of 50 °C/min to 285 °C and held at this level for 27.5 min. The GC was equipped with an HP-5 MS low bleed capillary column (30 m × 0.5 mm × 0.5 μm internal diameter; J&W, Folsom, CA), and helium was used as the carrier gas at a flow rate of 1.5 mL/min. The mass selective detective detector was kept at a temperature of 230 °C at the ion source and 180 °C at the quadruple. Electron impact (EI) mass spectra were obtained with an acceleration energy of 70 eV. Approximately 1.0 μL of the extract was injected in split/splitless mode using an autosampler 7683 from Agilent Technologies, Inc. Beijing, China. Fragment ions were analyzed in the mass range of 40–6000 *m*/*z* using full scan mode, with a filament delay time of 3 min. All parameters were integrated following the procedure.^31^ The data acquisition was performed using ChemStation (Agilent MSD ChemStation Data Analysis, F. 1. 0. 903).

FAMEs were identified by comparing their mass spectral data and retention times with those of authentic standards provided by the National Institute of Standards and Technology (NIST) 08 and 11 Library-MS databases. Relative quantification of the fatty acids was conducted by using the match criterion of >90%, and later, the ones identified were expressed as a percentage of the total molecules.

### Proximate Composition Analysis

The proximate analysis of the parameters dry matter, crude protein, crude fiber, fat, and ash content of the pig and rabbit manures as animal-derived substrates and potato and market wastes as plant-based substrates was determined according to AOAC.^32^ The reagents stated by Van Soest and colleagues were used to analyze acid detergent fiber (ADF) and neutral detergent fiber (NDF) with a Velp fiber analyzer (FIWE 6, VELP Scientifica, Usmate Velate, Italy).^33^ A mathematical equation was used to deduce carbohydrates from fat and protein, and total energy was calculated from the modified Atwater formula.^34^ For each treatment, three replicates were used.

### Statistical Analysis

Statistical analysis of data was performed by the one-way analysis of variance using R Ver. 4.2.1. The significant difference for each experimental group against the control was established by Turkey’s all-pairwise comparisons with a significance level of α = 0.05. The inhibition zones from disc diffusion tests were measured using ImageJ (1.5.3). Graphs were plotted using GraphPad Prism 8.0.1. 244. The nonmetric multidimensional scaling (NMDS) plots were prepared in a PAST program, and the linear correlation between variables was measured with Pearson’s correlation coefficient (r) in R Ver. 4.2.1.

## Results and Discussion

### Growth Performances of HIL on Different Organic Substrates

**Figure 1:**
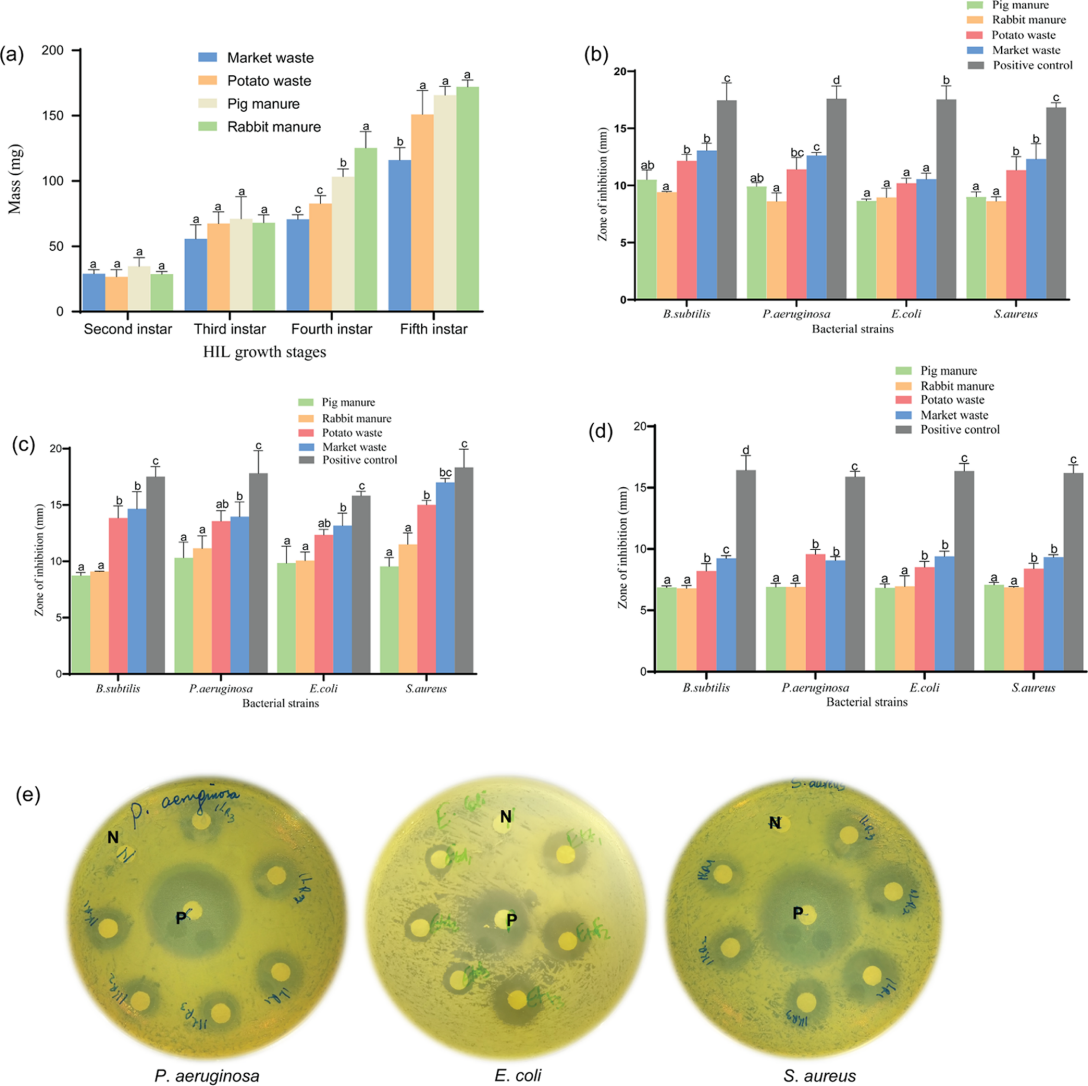
(a) Weight of *H. illucens* at different growth stages, (b) antibacterial activity of hexane extracts of *H. illucens* larvae, (c) antibacterial activity of 20% AcOH extracts of *H. illucens* larvae, and (d) antibacterial activity of 80% MeOH extracts of *H. illucens*. Different letters above each bar in individual groups of bars show statistical differences (Turkey contrasts, *P* < 0.001). (e) Representative photographs showing inhibitory zones of HIL extracts (2 mg/disc) against selected test pathogens *P. aeruginosa*, *E. coli*, and *S. aureus*.

Four organic substrates were collected to feed HIL to activate the innate immune system required for the production of antimicrobial molecules. Market and potato wastes were plant-based substrates, while the other two, pig and rabbit alimentary residues, were animal manures. The systematic growth of larvae from the second to fifth instar in all feeds was observed (Figure 1a and Table S2 in the Supporting Information). All substrates were used in unsterilized conditions. There is a possibility of sugars and amino acids being degraded by microorganisms and boosting larval development.^35^ In this study, it was found that the HIL fed on four different rearing substrates did not show a significant difference in the weights of individual larvae for second instar (df = 3, 8; *F* = 1.61; *P* = 0.262) and third instar (df = 3, 8; *F* = 1.043; *P* = 0.425; Figure 1a). Notably, animal-derived substrates were observed not to support the survivorship of the larvae. For instance, during harvesting, the weights of the larvae reared on plant-based substrates yielded 4008 g for market and 4200 g for potato waste, considerably higher than the counterpart larvae reared on animal-based substrates, 1600 g for pig and 950 g for rabbit manure. This observation tallies with the previous investigations, which found that plant-derived substrates (potato tubers, vegetables, and fruits) were excellent feeding media for the growth and development of HIL.^36^ This may be attributed to the presence of higher fats and digestible carbohydrates in plant-derived substrates, the primary source of energy for the growth and development of many insects,^37^ as opposed to animal-derived substrates. As a consequence, low competition for nutrients by the remaining larvae at the later stages of development (fourth and fifth instars) in animal-based substrates increased their weights than those of the counterparts in plant-based substrates where competition for food was higher (fourth instar df = 3, 8; *F* = 28.96; *P* = 0.00012; fifth instar df = 3, 8; *F* = 15.16; *P* = 0.00116; Figure 1a and Table S2 in Supporting Information). This finding corroborates previous report that the lesser the larval density, the lesser the competition for food.^38^

### Antibacterial Activity of HIL Extracted with Hexane

It was observed that the HIL dry biomass from market waste, pig manure, potato waste, and rabbit manure extracted with hexane showed antibacterial activities against all tested bacterial strains;*B. subtilis*,*S. aureus*,*P. aeruginosa*, and*E. coli* (Figure 1b,e and Table S3 in the Supporting Information).

The findings are consistent with past results indicating oily HIL extract from black soldier flies and yellow mealworms showed antibacterial efficacy against a variety of pathogens.^39^ There was a statistically significant difference in the antibacterial activity of extracts against test bacterial pathogens, as shown for *B. subtilis* (df = 4, 10; *F* = 43.78; *P* < 0.001), *S. aureus* (df = 4, 10; *F* = 89.35; *P* < 0.001), *P. aeruginosa* (df = 4, 10; *F* = 58.67; *P* < 0.001), and *E. coli* (df = 4, 10; *F* = 77.50; *P* < 0.001). This could suggest the presence of different metabolites and differential expression of bioactive metabolites by larvae reared on different substrates. Previous experiments demonstrate that*H. illucens*reared on different substrates exhibit accumulation of various genes responsible for vast spanning lipid^40^ and protein metabolism.^41^ Furthermore, transcriptome analyses of*H. illucens* have revealed genes that are responsible for different functions such as nutrition are expressed to varying levels in the midgut of larvae reared on a variety of food substrates.^42^ Owing to the paucity of information regarding the antimicrobial metabolites and their expression by HIL reared on different substrates, we sought to determine their influence on the antibacterial activities of HIL hexane extracts reared on different substrates. This study found that the extracts of the larvae reared on plant-based substrates had higher antibacterial activity than those reared on animal-based substrates. This suggests that the different substrates induced different metabolic pathways, thus producing an unequal index of metabolites.

### GC-MS Identification of Fatty Acids from HIL Hexane Extracts

Forty-seven fatty acids were detected from HIL fed on four organic substrates with retention times ranging from 14.82 to 31.97 min according to gas chromatography-mass spectrometry (GC-MS) data output (Table S1 in the Supporting Information). All extracts from the four substrates were found to have nine common methylated fatty acids (Table 1).

**Table 1:** Concentrations of Free Methylated Fatty Acids from HIL Extracted with Hexane^a^

	substrates for feeding HIL						
free methylated fatty acids	CH (μg/g)	MH (μg/g)	PH (μg/g)	RH (μg/g)	*F*-value	df	*P*-value
methyl nonanoate (**1**)	14.06 ± 5.49^a^	15.94 ± 12.94^a^	8.34 ± 0.44^a^	2.68 ± 0.57^a^	2.18	3, 8	>0.05
methyl undecanoate (**4**)	1.45 ± 0.66^a^	0.53 ± 0.13^a^	2.27 ± 0.64^ab^	3.97 ± 1.20^b^	11.11	3, 8	<0.01
methyl dodecanoate (**5**)	602.76 ± 88.94^b^	318.17 ± 174.18^ab^	214.42 ± 185.99^ab^	27.71 ± 9.78^a^	4.3	3, 8	<0.05
methyl hexadecanoate (**20**)	738.28 ± 42.68^c^	386.69 ± 123.30^b^	31.29 ± 21.16^a^	44.60 ± 6.72^a^	76.96	3, 8	<0.001
methyl docosanoate (**28**)	8.43 ± 2.03^a^	17.17 ± 6.85^a^	17.33 ± 14.39^a^	2.67 ± 0.51^a^	2.38	3, 8	>0.05
methyl 9*Z*-tetradecenoate (**34**)	39.10 ± 17.77^b^	18.22 ± 8.40^ab^	31.07 ± 17.77^ab^	1.11 ± 0.34^a^	5.42	3, 8	<0.05
methyl 10*Z*-heptadecenoate (**38**)	21.72 ± 6.19^ab^	30.33 ± 9.44^b^	19.11 ± 18.20^ab^	1.84 ± 1.15^a^	3.72	3, 8	>0.05
methyl (9*Z*,12*Z*)-octadecadienoate (**44**)	853.24 ± 154.78^b^	21.61 ± 4.56^a^	25.97 ± 18.99^a^	13.63 ± 5.09^a^	85.41	3, 8	<0.001
methyl (5*Z*,8*Z*,11*Z*,14*Z*)-eicosatetraenoate (**46**)	10.68 ± 3.12^a^	3.92 ± 1.01^a^	27.53 ± 22.07^a^	4.02 ± 1.55^a^	2.97	3, 8	>0.05

a HIL—*H. illucens* larvae, CH—potato waste-fed HIL extracted with hexane, MH—market waste-fed HIL extracted with hexane, PH—pig manure-fed HIL extracted with hexane, RH—rabbit manure-fed HIL extracted with hexane, letters on numbers represent the statistically significant difference between groups.

Methyl hexadecanoate (**20**) had considerably higher concentrations of 738.28 and 386.69 μg/g from market and potato wastes, respectively, whereas that of methyl dodecanoate (**5**) was 602.76 and 318.17 μg/g in , the lowest concentration of methyl dodecanoate (**5**) listed was 27.71 μg/g from HIL fed with the animal-based rabbit manure substrate. It is certain that the concentration of most SFAs was potentially increased by feeding the HIL with plant-based substrates, i.e., potato and market wastes.

**Figure 2:**
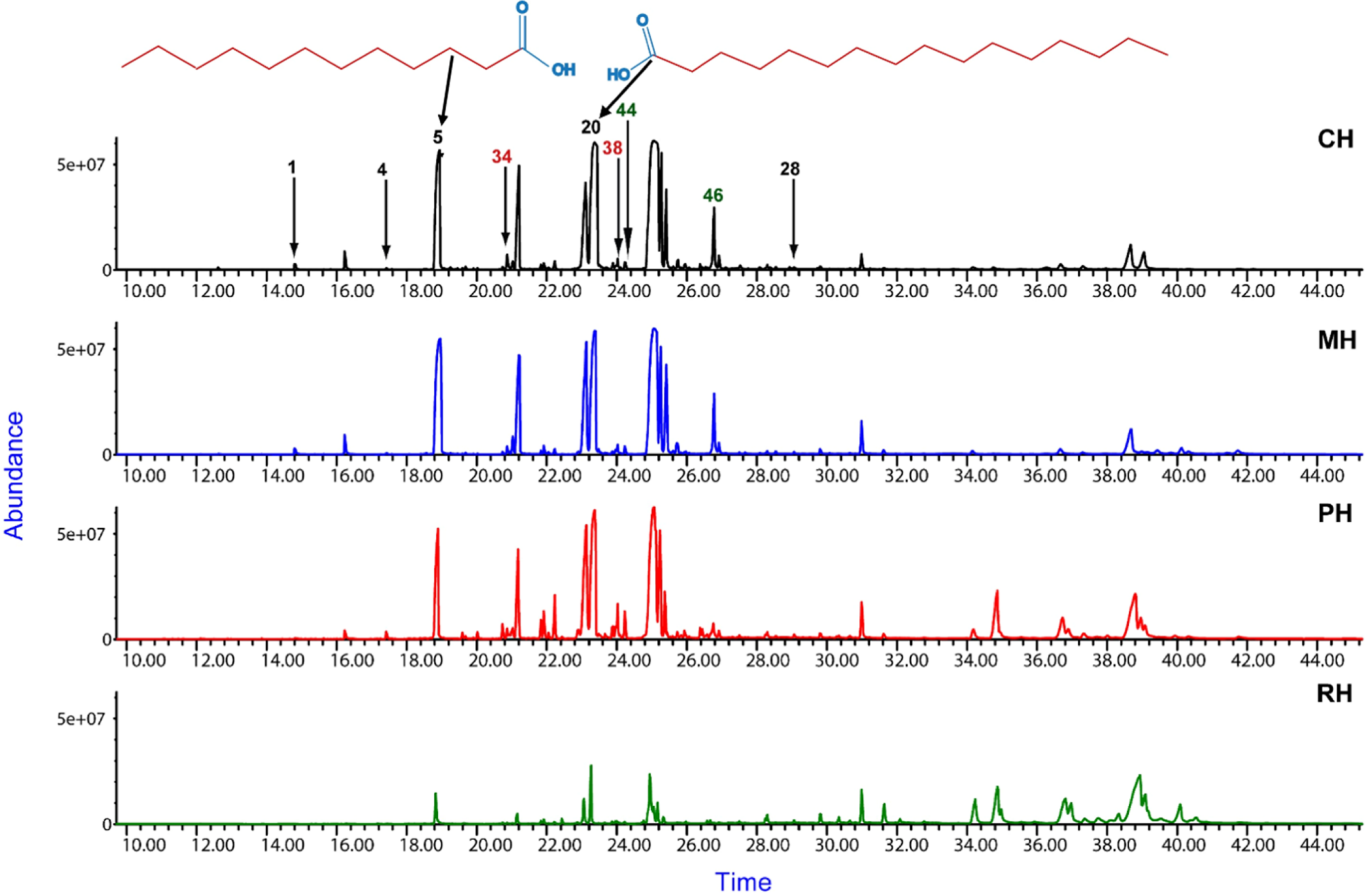
GC-MS spectrum of HIL extracts; CH = potato waste-fed HIL extracted with hexane, MH = market waste-fed HIL extracted with hexane, PH = pig manure-fed HIL extracted with hexane, and RH = rabbit manure-fed HIL extracted with hexane. The numbering of the identified common methyl esters in the four substrates is classified as follows: Black represents SFAs, red represents MUFAs, and green represents PUFAs; HIL,*H. illucens* larvae.

Substantial quantities of 39.10 μg/g were registered for methyl 9*Z*-tetradecenoate (**34**) reared on market waste with the lowest value of 1.11 μg/g recorded from HIL fed with rabbit manures. An incredible level, 853.24 μg/g methyl (9*Z*,12*Z*)-octadecadienoate (**44**), one of the most common polyunsaturated fatty acids (PUFAs) in living systems, was discerned in extracts from market waste-fed HIL with lower concentrations of 13.63 μg/g recorded on extracts from rabbit manure-fed HIL. These profiles are reminiscent of the previous reports portraying dodecanoic acid, hexadecenoic acid, 9*Z*-tetradeccenoic acid, 9*Z*,12*Z* octadecadienoic acid, and 5*Z*,8*Z*,11*Z*,14*Z*-eicosatetraenoic acid as the dominant fatty acids in HIL extracts.^43^ The total ion chromatograms of fatty acids obtained from GC-MS representing the common fatty acids in all four extracts are shown in Figure 2.

Methyl dodecanoate (**5**) and methyl hexadecenoate (**20**) with retention times of 18.92 and 23.37 min, respectively, were expressed as clear peaks in all four extracts, whereas methyl-(5*Z*,8*Z*,11*Z*,14*Z*)-eicosatetraenoate (**46**), with a retention time of 26.40 min, showed a coherent peak in only potato and market waste extracts. Methyl nonanoate (**1**), methyl docosanoate (**27**), and methyl (9*Z*,12*Z*)-octadecadienoate (**44**) indicated trifling peaks at retentions times of 14.82, 28.55, and 24.4 7 min, respectively. These findings are consistent with documented data on HIL fed with kitchen waste, chicken manure, and brewers’ spent grain.^44^

Our experiments identified dodecanoic acid (*viz*. lauric acid), a known active component in antibacterial activity that acts by the spatial arrangement of atoms disrupting membranes.^45^ In the same study, lauric acid was found to have greater antibacterial action than capric acid, which has two fewer carbons than lauric acid. Based on its chemical structure, it is certain that it has hydrophilic properties, brought about by the presence of the –OH group and the O atom of the carbonyl group (blue in color, Figure 2). Both groups form H-bonds with the polar part of the cell walls of pathogenic Gram-positive microorganisms, an interaction that consequently disrupts the bacterial cell membrane.^45^ Meanwhile, the lauryl group (red in color, Figure 2), which potentially forms van der Waals interactions with a negative envelope of Gram-negative bacteria, contributes to lipophilic properties.^46,47^ This is in agreement with the findings that the lauryl part, in the case of lauric acid, penetrates the negative bacterial membrane and disrupts the cell wall by physicochemical processes, leading to the death of Gram-negative bacteria.^48^ This could also be applied to our study as registered for the antibacterial activities of HIL extracts against*E. coli* and*P. aeruginosa*. The increased antibacterial action of HIL extracts from market and potato wastes against*S. aureus*and*P. aeruginosa*could be attributed to their metabolic profile where lauric and palmitic acids were overexpressed.

**Figure 3:**
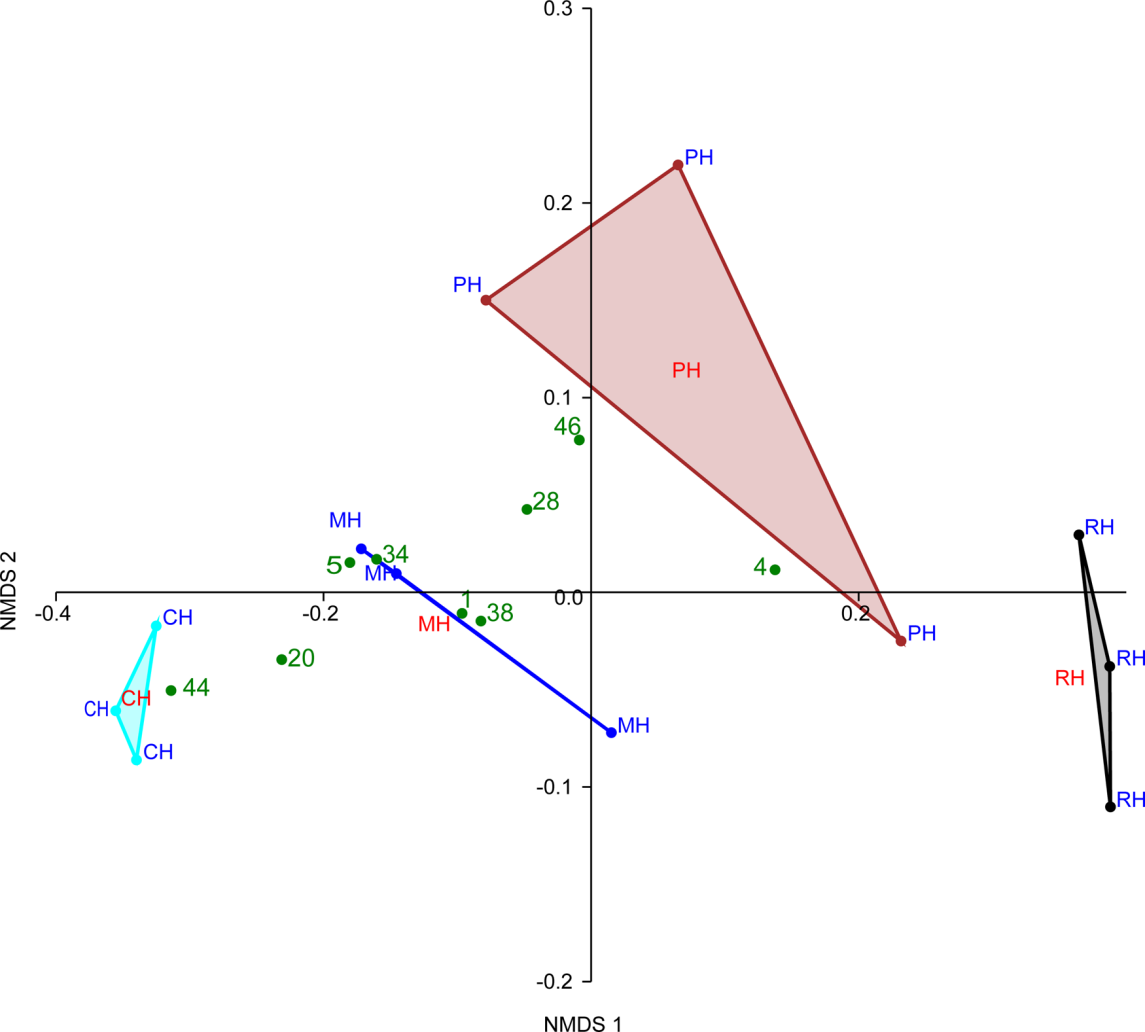
NMDS clustering of samples of HIL fed with MH, market waste; CH, potato waste; RH, rabbit manure, and PH, pig manure, and extracted with hexane based on nine common free fatty acids in samples. The identity of free fatty acids with their numbers is in Table 1.

The results of nonmetric multidimensional scaling (NMDS) analysis provide valuable insights into the understandable output of fatty acids from the four distinct extracts of HIL, as shown in Figure 3.

The NMDS plot shows that of the nine common fatty acids identified, eight fell in the upper and lower quadrant of NMDS. Two bioactive methylated fatty acids, methyl dodecanoate (**5**) and methyl hexadecenoate (**20**), were strongly associated with HIL fed with market and potato waste substrates, further confirming the influential effects of feeding substrates on the fatty acids’ spectra of HIL.^49^

Having determined the fatty acids’ profile and their distribution in substrates, it was distinct that the antibacterial activity of the extracts could be largely due to the presence of *n*-dodecanoic acid (**5**) and hexadecanoic acid (**20**), commonly referred to as lauric acid and palmitic acid, respectively. These two are considered responsible for antibacterial activity in oily extracts from HIL samples, a finding that agrees with the previous report.^50^ The two fatty acids have their concentrations higher in HIL fed with market waste and potato waste. This observation concurs with the previous study, which reiterated that*H. illucens*larvae reared in an environment that resulted in higher larval density had their phenol-oxidase enzyme increased.^51^ Phenol-oxidase has an increased role in an insect-sufficient immune response, which in turn increases the antibacterial activities of the larval extracts, as documented in this study.

### Antibacterial Activities of 20% AcOH and 80% MeOH Extracts from HIL

The antibacterial activity of 20% acetic acid extracts of HIL varied between the animal- and plant-based rearing substrates. Against each test pathogen, extracts of HIL reared on plant-derived wastes were significantly more active (12.35–17.00 mm) than those reared on animal-based wastes (8.73–11.50 mm; *P* < 0.05). There was no significant difference in antibacterial activity between plant-based substrates (market and potato wastes) and animal-derived wastes (pig and rabbit manures) when compared (*P* > 0.05; Figure 1c and Table S4 in the Supporting Information). The 20% AcOH extracts from HIL fed with market waste, pig manure, potato waste, and rabbit manure showed relatively higher antibacterial activities ranging from 8.73 to 17.00 mm than their counterpart from 80% MeOH extracts (6.80–9.25 mm). This may be attributed to the difference in solvent polarities and consequently the type of metabolites extracted. Polar solvents are more amenable to extracting hydrophilic metabolites, whereas hydrophobic solvents extract nonpolar metabolites.^52,53^ The solution of 20% AcOH is known to be suitable for the extraction of peptides and polypeptides.^14^ This might explain why its extracts exhibited significantly higher antibacterial activities. Peptides and polypeptides are an important part of innate immunity in microorganisms, humans, and animals.^54,55^ Importantly, peptides and polypeptides are reported to possess vast biological activities including immune regulation, antiaging, anticancer activities, and antibacterial activities.^56^ The known mechanism of action of peptides and polypeptides as antibacterial agents is that they act against bacteria by forming pores in the cell membrane, thereby disrupting it and causing cell death.^57^ Notably, in line with this study, HIL reared on plant-based substrates and extracted with 20% AcOH exhibited elevated antibacterial activities than those reared on animal-based extracts. Feeding*H. illucens*larvae with the fruit, vegetable, and potato waste substrate could have ignited the expression of antimicrobial peptide genes that enhance the level of AMPs present in the extracts, thus leading to better antibacterial activity. On the other hand, 80% MeOH extracts were observed to have lower antibacterial activities than 20% AcOH. This could be due to the ability of 80% methanol solvent to extract a wider range of metabolites such as phenols, polyphenols, terpenes, sesquiterpenes, and alkaloids.^58^ Some of these molecules may not have antibacterial activities comparable to peptides and polypeptides.^59^ The non-hydrogen forces contained in 80% MeOH interact with test pathogens through hydrophobic interactions or covalent bonding.^60^ These forces of attraction may not be sufficient to break the bacterial membranes, hence the reduced antibacterial activities in these extracts.^61^ Additionally, the better antibacterial activities in plant-based substrates recorded in 80% methanolic extracts (Figure 1d and Table S5 in the Supporting Information) could be a result of feeding*H. illucens*larvae with substrates that have higher fat, energy, and carbohydrate content, leading to overexpression of phenolic compounds and enhancing the antibacterial activity. For instance, phenols show antibacterial activity against a variety of bacterial strains through various modes of action, including membrane damage,^62^ enzyme inhibition,^63^ bacterial metabolic change,^63^ and shattering of bacterial DNA.^64^ The comparison of the three extracts from hexane, 20% acetic acid, and 80% methanol revealed 20% AcOH extracts to have superior antibacterial activities and thus were subjected to serial dilution assays.

### Minimum Inhibitory Concentration (MIC) and Minimum Bactericidal Concentration (MBC) Assays

The MIC and MBC values of HIL extracts with 20% AcOH from plant-derived substrates of market and potato wastes and animal-based substrates of pig and rabbit manures were recorded to be 25 mg/mL across all of the test bacterial pathogens (Table 2).

**Table 2:** MIC and MBC Values for Extracts from HIL Fed with Different Organic Substrates^a^

tested extracts	concentration (mg/mL)	*B. subtilis*	*P. aeruginosa*	*S. aureus*	*E. coli*
PAcE	MIC	25 ± 00	25 ± 00	25 ± 00	25 ± 00
	MBC	50 ± 00	50 ± 00	50 ± 00	50 ± 00
RAcE	MIC	25 ± 00	25 ± 00	25 ± 00	25 ± 00
	MBC	50 ± 00	50 ± 00	50 ± 00	50 ± 00
CAcE	MIC	25 ± 00	25 ± 00	25 ± 00	25 ± 00
	MBC	25 ± 00	25 ± 00	25 ± 00	25 ± 00
MAcE	MIC	25 ± 00	25 ± 00	25 ± 00	25 ± 00
	MBC	25 ± 00	25 ± 00	25 ± 00	25 ± 00

a MIC—minimum inhibitory concentration, MBC—minimum bactericidal concentration, AcOH—acetic acid. PAcE, pig manure HIL extracted with 20% AcOH; RAcE, rabbit manure HIL extracted with 20% AcOH; CacE, potato waste HIL extracted with 20% AcOH; MAcE, market waste HIL extracted with 20% AcOH; HIL, *H. illucens* larave.

This could present an abundance of AMPs in the extract from plant-based substrates richer in total energy, fat, and carbohydrate, thus inhibiting the growth of the bacterial population to the same concentration as evident through MIC and MBC. Nevertheless, the MBC values for the extracts derived from animal manure substrates were observed at 50 mg/mL, and MIC remains at 25 mg/mL for all test pathogens. The lesser expression of AMP genes in animal-based extracts could allow the test microorganism to recover from HIL extracts and grow further,^50^ thus leading to higher MBC values. It was equally noted that the MBCs of HIL extracts as found in the current investigation were lower than 320 mg/mL of HIL extracts in a prepupae stage as formerly reported.^65^

### Time-Kill Assays

**Figure 4:**
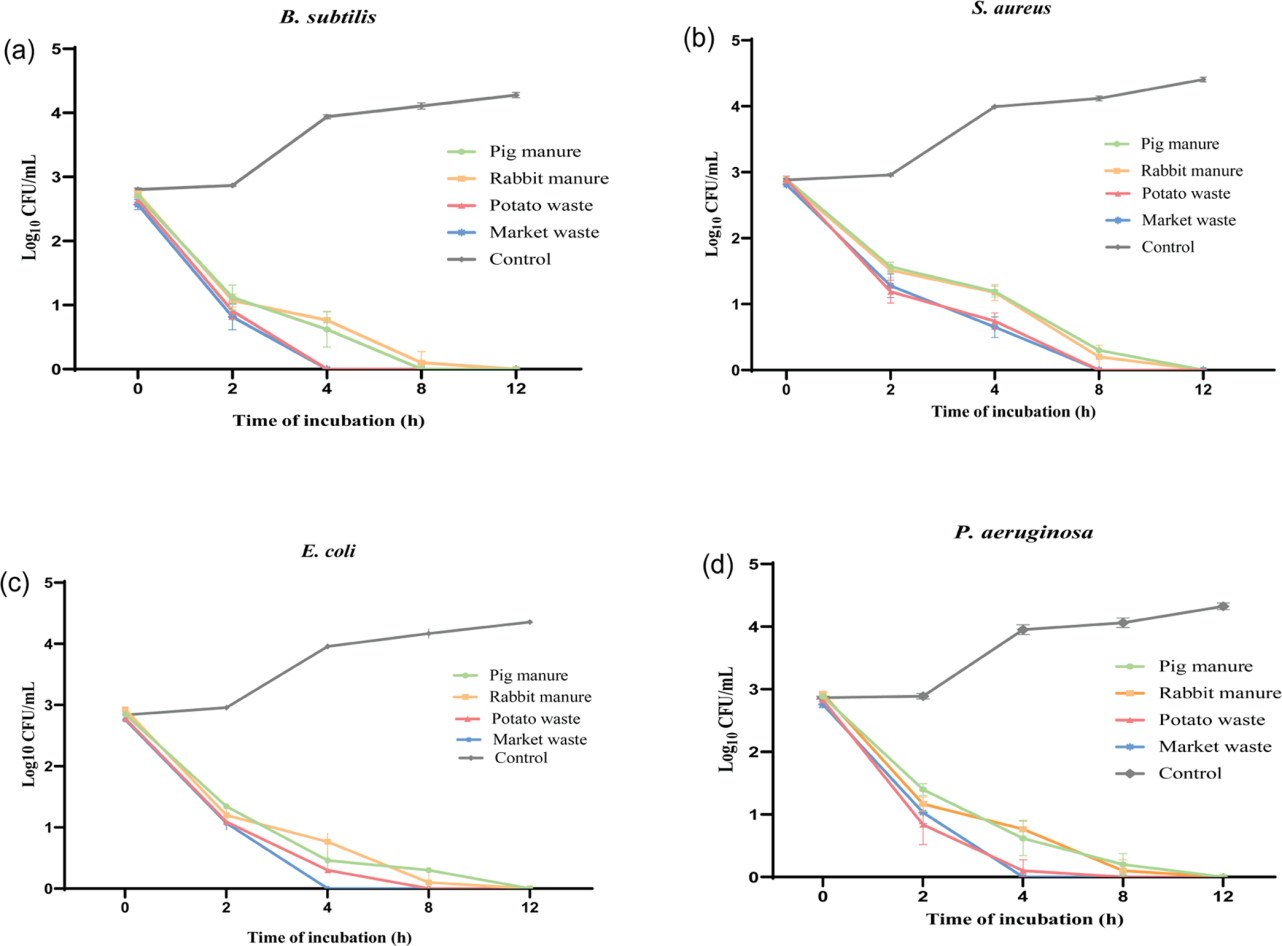
Time-kill curve of HIL fed with organic substrates and extracted with 20% acetic acid, market waste, MIC (25 mg/mL), potato waste, MIC (25 mg/mL), rabbit manure, MIC (25 mg/mL), and pig manure, MIC (25 mg/mL) against (a)*B. subtilis,* (b)*S. aureus,* (c)*E. coli,* and (d)*P. aeruginosa*.

The time-kill assay curves were plotted based on the MIC results obtained from each test pathogen using the 20% AcOH extracts (Figure 4 (a–d)).

For the positive control (0 mg/mL; no extract), an exponential increment in bacterial population was observed from 0 to 12 h in all four tested bacterial species. These results revealed that the activity of plant-based extracts from*H. illucens* against Gram-negative bacteria*E. coli* and*P. aeruginosa*, as well as Gram-positive bacteria*B. subtilis*, resulted in the death of the bacterial population at the fourth hour. Considering the set range of incubation conditions, this is the earliest time determined for the activity of extracts against test pathogens. This could be attributed to the nature of AMPs present in the extracts, which manifested themselves strongly in plant-based extracts, leading to the early death of the bacterial population.^66^

Additionally, HIL extracts from plant-based substrates when tested against*S. aureus* dragged up to the eighth hour for complete depletion of the bacterial colonies to be observed. The precedent result recorded could be a result of the structural nature of*S. aureus*, a Gram-positive bacterium that is known to develop resistance, which is manifested in such a way that it produces a capsule-like coating of polysaccharides that protects its cell wall against antimicrobial drugs.^67^ Furthermore, the extracts from HIL fed with animal-based substrates had an effect at the 12th hour, the time that displayed the whole population of bacteria dead. The longer time taken by HIL extracts from animal-based substrates to destroy the bacterial population might be a result of lower AMP expression in these extracts.^50^ Overally, the trend observed for the these assays was time-dependent.^29^

### Proximate Composition Analysis and Pearson’s Correlation

The proximate analysis of the potato and market wastes as plant-based substrates and pig and rabbit manures as animal-derived feeds for*H. illucens* larvae showed statistically significant values for all of the parameters tested except for carbohydrate content (Table 3).

**Table 3:** Proximate Composition of Four Organic Substrates^a^

sample	% DM	ASH (% DM)	CFAT (% DM)	CFIBR (% DM)	ADF (% DM)	NDF (% DM)	CP (% DM)	CHO (% DM)	ENGY (kcal/100 g DM)
RMS	93.5^b^ ± 0.00	13.0^c^ ± 0.62	1.1^a^ ± 0.00	1.0^c^ ± 0.16	46.7^c^ ± 0.61	67.7^d^ ± 0.62	27.8^b^ ± 0.50	50.4^a^ ± 0.62	323.3^b^ ± 2.09
CWS	88.3^a^ ± 0.58	5.1^a^ ± 0.03	14.3^c^ ± 0.74	0.3^a^ ± 0.03	15.1^a^ ± 1.63	41.4^a^ ± 0.62	14.1^a^ ± 2.50	54.5^a^ ± 2.67	403.5^c^ ± 1.39
PMS	96.8^c^ ± 0.29	24.3^d^ ± 0.45	2.4^a^ ± 0.59	0.7^b^ ± 0.04	42.7^c^ ± 1.55	61.3^c^ ± 1.23	15.5^a^ ± 2.11	53.9^a^ ± 2.51	299.6^a^ ± 1.75
MWS	93.0^b^ ± 0.00	11.1^b^ ± 0.31	11.1^b^ ± 0.62	0.7^b^ ± 0.06	36.2^b^ ± 2.48	44.9^b^ ± 1.07	15.1^a^ ± 1.11	55.1^a^ ± 1.16	466.3^d^ ± 4.28
*P*-values	<0.001	<0.001	<0.001	<0.001	<0.001	<0.001	<0.001	0.07	<0.001
*F*-values	353.4	1120	392.1	32.87	204.6	564.2	42.31	3.37	2532
df	3, 8	3, 8	3, 8	3, 8	3, 8	3, 8	3, 8	3, 8	3, 8

a Means (± standard deviation) of proximate composition (in % dry matter) of four organic substrates used to rear black soldier fly larvae. Means (*n* = 3) are significantly different at *P* < 0.05. Different superscript letters represent the statistical significance. RMS, rabbit manure substrate; CWS, potato waste substrate; PMS, pig manure substrate; MWS, market waste substrate; DM, dry matter; CFAT, crude fat; CFIBR; ADF, acid detergent fiber; NDF, neutral detergent fiber; CP, crude protein; CHO, carbohydrates; and ENGY, total energy.

When examining the plant-based substrates, it is evident that they possess higher crude fat (CFAT, *F* = 392.1; df = 3,8; *P* < 0.001) content than the animal-based substrates (Table 3). The elevated crude fat (CFAT) content of the substrate enhances the*H. illucens*larval growth.^68^ Additionally, plant-based substrates were observed to exhibit higher ash (*F* = 1120; df = 3,8; *P* < 0.001) content accompanied by greater total energy (ENGY, *F* = 2532; df = 3,8; *P* < 0.001) as evident in Table 3 for optimum larval growth. On the contrary, animal-based substrates showed higher levels of acid detergent fiber (ADF, *F* = 204.6; df = 3,8; *P* < 0.001) and neutral detergent fiber (NDF, *F* = 564.2; df = 3,8; *P* < 0.001).

**Figure 5:**
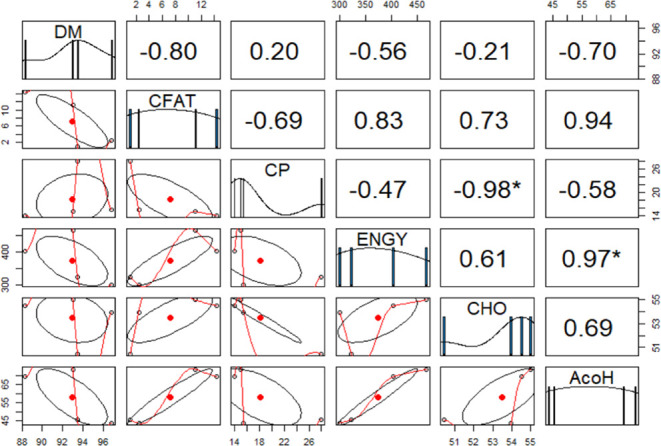
Pearson’s correlation coefficient (*r*); correlations for proximate analysis and antibacterial activity. AcOH, antibacterial activity of 20% acetic acid extract; DM, dry matter; CFAT, crude fat; CFIBR; ADF, acid detergent fiber; NDF, neutral detergent fiber; CP, crude protein; CHO, carbohydrates; and ENGY, total energy.

Interestingly, there was a strong correlation between the total energy content (ENGY) and antibacterial activity (AcOH; *r* = 0.97*), as shown in Figure 5.

The other positive correlations observed were in crude fat (CFAT) and antibacterial activity (*r* = 0.94), carbohydrate content and antibacterial activity (*r* = 0.69), total energy and crude fat content (CFAT; *r* = 0.83), and carbohydrate and total energy (*r* = 0.61; Figure 5). Conversely, a negative correlation was observed between crude protein (CP) and antibacterial activity (*r* = −0.58), CP and total energy content (*r* = −0.47), and CP and the carbohydrate level (*r* = −0.98*; Figure 5). Similarly, positive correlation values for crude fat content, carbohydrate, and total energy were obtained in the case of hexane extract with antibacterial activity (Figure S1 in the Supporting Information). These findings suggest that factors that influence the antibacterial activities of the*H. illucens* larval extracts include fat, total energy, and to some extent carbohydrate content in the extracts. The latter argument is supported by a positive correlation between antibacterial activity and fat, total energy content, and carbohydrates. The composition of the larval extracts is observed to result from the nutrient richness of the substrates on which they are fed. This is demonstrated by the fact that*H. illucens* larvae fed with high-fat substrates create a rich fatty acid profile.^69^ Furthermore, it is confirmed that plant-based substrates with increased energy content endowed HIL extracts with stronger antibacterial activity.

## Conclusions

This study found that extracts from HIL fed with carbohydrate and fat-rich substrates such as market and potato waste produced higher energy content and had better antibacterial activities. The level of antibacterial activities increased in HIL extracts from plant-based substrates obtained with the three solvents used. This study therefore provides evidence that the antibacterial activities of extracts can be influenced by the substrate in which HIL are fed. These findings pave the way for the possible use of market and potato waste as substrates for rearing HIL, which is necessary for producing antimicrobial metabolites. Moreover, the utilization of plant-based wastes as substrates will not only reduce the pollution of the environment, since they are easily biodegradable but also open the way to controlling antimicrobial resistance. The antibacterial activity of HIL hexane extracts could be attributed to the presence of lauric acid in appreciable concentrations. Future perspectives may focus on the isolation of the individual bioactive agents responsible for observed antibacterial activity in HIL extracts targeting mostly the AMPs extracted with 20% AcOH.

## Supporting Information Available

Pearson’s correlation coefficient (*r*) for proximate analysis and antibacterial activity of hexane extract (Figure S1); concentrations of methylated fatty acid from GC-MS (Table S1); mass of HIL in different growth stages (Table S2); antibacterial activity of HIL extracted with hexane (Table S3); antibacterial activity of HIL extracted with 20% acetic acid (Table S4); and antibacterial activity of HIL extracted with 80% methanol (Table S5) (PDF)

## Notes

Ethical Statement: The authority to conduct the experiment and collect data was in accordance with the animal welfare regulations and granted by the National Commission for Science, Technology, and Innovation (NACOSTI); Research Permit License No: NACOSTI/P/23/29359. This research also received approval from the Institutional Animal Care and Use Committee (IACUC) of Kenya Agricultural and Livestock Research Organization (KALRO)-Veterinary Science Research Institute (VSRI); Muguga North upon compliance with all provisions vetted under and coded: KALRO-VSRI/IACUC028/16032022.
