# Supplementary material for: Unlocking the Potential of Substrate Quality for the Enhanced Antibacterial Activity of Black Soldier Fly against Pathogens
Source: ACS Omega. 2024 Feb 12;9(7):8478–89. doi: 10.1021/acsomega.3c09741 (PMC10882654; doi:10.1021/acsomega.3c09741)
Supplement: Supplementary file 1 — ao3c09741_si_001.pdf [file ao3c09741_si_001.pdf]

Unlocking the Potential of Substrate Quality for Enhanced Antibacterial Activity of Black Soldier Fly against Pathogens

Mach P. Achuoth[1,2,3], Cynthia M. Mudalungu[1,4]*, Brian O. Ochieng[1], Hosea O. Mokaya[1], Shadrack Kibet[1], Vinesh J. Maharaj[2], Sevgan Subramanian[1], Segenet Kelemu[1] and Chrysantus M. Tanga[1]

[1]International Centre of Insect Physiology and Ecology, P. O. Box 30772-00100, Nairobi, Kenya

[2]Department of Chemistry, Faculty of Natural and Agricultural Sciences, University of Pretoria, Private bag x20, Hatfield, 0028, South Africa

[3]Department of Chemistry, College of Science and Technology, Dr John Garang Memorial University of Science and Technology-Bor, P. O. Box 436 Juba, South Sudan

[4]School of Chemistry and Material Science, The Technical University of Kenya (TUK), P.O Box 52428 - 00100, Nairobi – Kenya

*Correspondence: cmudalungu@icipe.org

Supporting Information

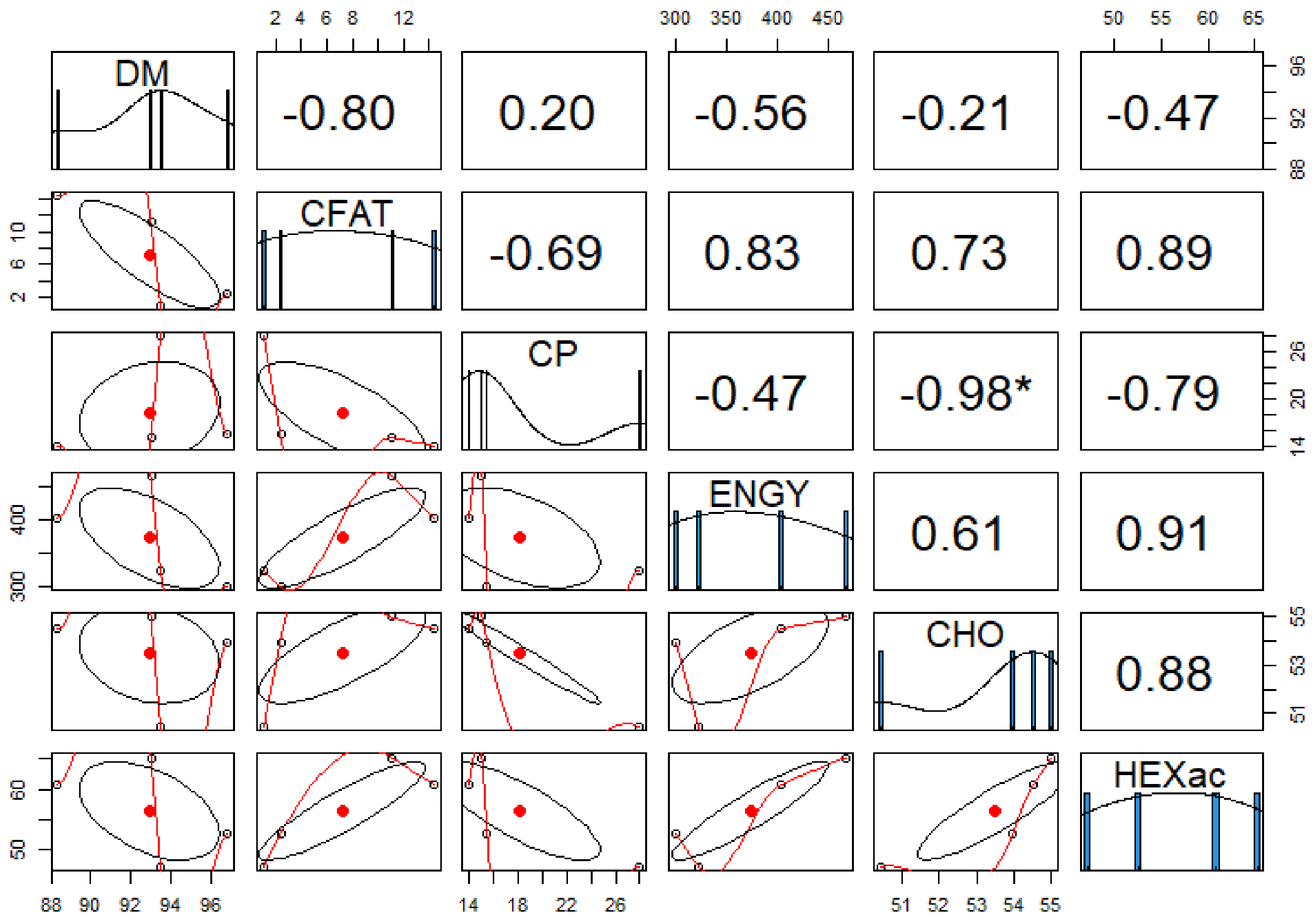

Supplementary Figure S1. Pearson's correlation coefficient (r) for proximate analysis and antibacterial activity of hexane extract.

Supplementary Table S1. Concentration of methylated fatty acids from hexane extracts of *H. illucens* larvae fed on CH, potato waste; MH, market waste; PH, pig manure and RH, rabbit manure.

S/NO	RT	Library/ID	Mean CH (µg/g)	Mean_MH (µg/g)	Mean PH (µg/g)	Mean_RH (µg/g)	F	DF	P- VALUE
1	14.82	Methyl nonanoate	14.06 ± 5.49[a]	15.94 ± 12.94[a]	8.34 ± 0.44[a]	2.68 ± 0.57[a]	2.18	3,8	> 0.05
2	16.25	Methyl decanoate	28.90 ± 9.73 [a]	30.89 ± 20.02[a]	33.54 ± 3.17[a]	-	0.1	2,6	> 0.05
3	16.97	Methyl 4,8-dimethylnonanoate	0.84 ± 0.13[ab]	0.18 ± 0.09[a]	1.53 ± 0.71[b]	-	7.65	2,6	0.05
4	17.57	Methyl undecanoate	1.45 ± 0.66[a]	0.53 ± 0.13[a]	2.27 ± 0.64[ab]	3.97 ± 1.20[b]	11.11	3,8	0.01
5	18.92	Methyl dodecanoate	602.76 ± 88.94[c]	318.17 ± 174.18[ab]	214.42 ± 185.99[ab]	27.71 ± 9.78[a]	4.3	3,8	0.05
6	19.25	Methyl 2-methyl-dodecanoate	4.29 ± 0.79	-	-	-			
7	19.27	Methyl 2,6-dimethyl-undecanoate	3.870 ± 1.58	-	-	-			
8	19.30	Methyl dodecanoate	-	-	1.12 ± 0.21	-			
9	19.45	Methyl 4-methyl-dodecanoate	2.94 ± 0.77[a]	2.35 ± 1.52[a]	-	-	0.36	1,4	> 0.05
10	19.61	Methyl 11-methyl-dodecanoate	-	-	2.01 ± 1.28	-			
11	19.72	Methyl 10-methyl-dodecanoate	-	3.37 ± 2.11	-	-			
12	20.46	Methyl 3-methyl-tridecanoate	-	0.51 ± 0.09[a]	0.51 ± 0.02[a]	-	0.0002	1,4	> 0.05
13	21.16	Methyl tetradecanoate	-	-	214.91 ± 94.05[b]	11.92 ± 1.94[a]	13.97	1,4	0.05
14	21.51	Methyl 2-methyl-dodecanoate	3.31 ± 0.59[a]	2.64 ± 0.45[a]	-	-	2.38	1,4	> 0.05
15	21.72	Methyl 12-methyl-tetradecanoate	4.39 ± 1.43[a]	26.00 ± 20.28[a]	-	-	3.39	1,4	> 0.05
16	21.94	Methyl 9-methyltetradecanoate	3.99 ± 1.74[a]	2.36 ± 2.24[a]	-	-	0.99	1,4	> 0.05
17	21.94	Methyl pentadecanoate	22.46 ± 18.38	-	-	-			
18	22.23	Methyl dodecanoate	-	-	59.41 ± 46.81	-			
19	22.61	Methyl 3-methyl-pentadecanoate	-	-	-	0.38 ± 0.06			
20	23.37	Methyl hexadecanoate	738.28 ± 42.68[c]	386.69 ± 123.30[b]	31.29 ± 21.16[a]	44.60 ± 6.72[a]	76.96	3,8	0.001
21	23.93	Methyl 15-methyl-hexadecanoate	12.02 ± 1.20[b]	4.04 ± 1.22[a]	-	-	64.97	1,4	0.01
22	23.96	Methyl heptadecanoate	-	-	-	6.15 ± 0.19			
23	25.52	Methyl nonadecanoate	-	-	3.25 ± 1.03	-			
24	26.93	Methyl 18-methylnonadecanoate	-	-	11.94 ± 2.26[b]	3.76 ± 1.16[a]	30.99	1,4	0.01

25	27.74	Methyl 18-methylicosanoate	-	-	-	3.05 ± 1.43			
26	27.76	Methyl heneicosanoate	-	-	4.11 ± 0.96	-			
27	28.31	Methyl octadecanoate	2.92 ± 1.37[a]	5.39 ± 0.20[a]	1.56 ± 1.97[a]	-	1.18	2,6	
28	28.55	Methyl docosanoate	8.43 ± 2.03[a]	17.17 ± 6.85[a]	17.33 ± 14.39[a]	2.67 ± 0.51[a]	2.38	3,8	> 0.05
29	29.31	Methyl tricosanoate	3.72 ± 1.34[a]	4.29 ± 0.30[a]	2.56 ± 2.05[a]	-	1.13	2,6	> 0.05
30	30.09	Methyl tetracosanoate	4.07 ± 1.17[a]	5.12 ± 1.33[a]	3.67 ± 2.08[a]	-	0.67	2,7	> 0.05
31	31.97	Methyl hexacosanoate	2.53 ± 0.85[a]	2.63 ± 0.60[a]	2.53 ± 0.19[a]	-	0.03	2,8	> 0.05
		Σ SFA	1262.31	822.88	614.74	106.89			
32	17.46	Methyl 3Z-octenoate	-	-	-	0.42 ± 0.14			
33	18.56	Methyl 5Z-dodecenoate	2.78 ± 0.31a	-	2.33 ± 0.91[a]	-	0.64	1,4	> 0.05
34	20.89	Methyl 9Z-tetradecenoate	39.10 ± 17.77[b]	18.22 ± 8.40a[b]	31.07 ± 17.77[ab]	1.11 ± 0.34[a]	5.42	3,8	0.05
35	20.91	Methyl 5Z-dodecenoate	-	32.50 ± 27.02	-	-			
36	22.07	Methyl 7Z-hexadecenoate	-	-	-	1.09 ± 0.36			
37	23.22	Methyl 9Z-hexadecenoate	-	-	550.42 ± 124.99[b]	50.58 ± 5.19[a]	47.89	1,4	0.01
38	24.04	Methyl 10Z-heptadecenoate	21.72 ± 6.19[ab]	30.33 ± 9.44[b]	19.11 ± 18.20[ab]	1.84 ± 1.15[a]	3.72	3,8	> 0.05
39	25.07	Methyl 9E-octadecenoate	-	2.22 ± 2.30[a]	268.67 ± 221.10[a]	115.25 ± 21.83[a]	3.26	2,6	> 0.05
40	25.45	Methyl 10-octadecenoate	-	21.04 ± 10.97	-	-			> 0.05
41	25.88	Methyl 10Z-nonadecenoate	4.22 ± 1.17[a]	17.89 ± 9.67[a]	19.15 ± 23.78	-	5.9	1,4	> 0.05
42	26.11	Methyl 2-heptenoate	-	18.75 ± 7.97	-	-			
43	26.96	Methyl 11-eicosenoate	-	19.58 ± 10.93	-	-			
		Σ MUFA	67.82	160.53	890.75	170.29			
44	24.47	Methyl (9Z,12Z)-octadecadienoate	853.24 ± 154.78[b]	21.61 ± 4.56[a]	25.97 ± 18.99[a]	13.63 ± 5.09[a]	85.41	3,8	0.001
45	25.61	Methyl 8,11,14-eicosatrienoate	3.48 ± 0.29	-	-	-			
46	26.40	Methyl (5Z,8Z,11Z,14Z)-eicosatetraenoate	10.68 ± 3.12[a]	3.92 ± 1.01[a]	27.53 ± 22.07[a]	4.02 ± 1.55[a]	2.97	3,8	> 0.05
47	26.53	Methyl 7,10,13-eicosatrienoate	10.23 ± 1.46	-	-	-			
		Σ PUFA	877.63	25.53	53.50	17.65			
		PUFA/SFA	0.70	0.31	0.09	0.16			

Supplementary Table S2. Mass of HIL (mg) in growth stages

Growth stages	Rabbit manure	Potato waste	Pig manure	Market waste	DF	F-Value	P-Value
Second instar	$28.7^{a} \pm 2.1$	$26.7^{a} \pm 5.5$	$34.7^{a} \pm 6.6$	$29.0^{a} \pm 3.0$	3, 8	1.61	0.262
Third instar	$68.0^{a} \pm 6.0$	$67.3^{a} \pm 9.0$	$71.0^{a} \pm 17.0$	$55.7^{a} \pm 10.8$	3, 8	1.043	0.425
Fourth instar	$125.3^{a} \pm 12.5$	$82.7^{a} \pm 6.0$	$103.3^{b} \pm 5.8$	$70.7^{a} \pm 3.5$	3, 8	28.96	0.00012
Fifth instar	$172.0^{a} \pm 5.3$	$151.0^{a} \pm 18.2$	$165.7^{a} \pm 6.7$	$116.0^{b} \pm 9.5$	3, 8	15.16	0.00116

Supplementary Table S3. Antibacterial activities of HIL Hexane extracts

Test pathogens	Rabbit manure	Potato waste	Pig manure	Market waste	Positive control	DF	F-Value	P-Value
B. subtilis	$9.41^{a} \pm 0.07$	$12.16^{b} \pm 0.56$	$10.51^{ab} \pm 0.84$	$13.04^{b} \pm 0.63$	$17.47^{c} \pm 1.50$	4, 10	43.78	<0.001
P. aeruginosa	$8.61^{a} \pm 0.74$	$11.41^{bc} \pm 1.05$	$9.92^{ab} \pm 0.33$	$12.63^{c} \pm 0.26$	$17.60^{d} \pm 1.11$	4, 10	58.67	<0.001
E. coli	$8.95^{a} \pm 0.81$	$10.20^{a} \pm 0.44$	$8.66^{a} \pm 0.16$	$10.56^{a} \pm 0.52$	$17.53^{b} \pm 1.19$	4, 10	77.5	<0.001
S. aureus	$8.62^{a} \pm 0.39$	$11.34^{ab} \pm 1.18$	$9.00^{a} \pm 0.44$	$12.31^{b} \pm 1.35$	$16.83^{c} \pm 0.42$	4, 10	89.35	<0.001

Supplementary Table S4. Antibacterial activities of HIL 20 % Acetic acid extracts

Test pathogens	Rabbit manure	Potato waste	Pig manure	Market waste	Positive control	DF	F-Value	P-Value
B. subtilis	9.10[a] ± 0.03	13.85[b] ± 1.07	8.73[a] ± 0.27	14.66[b] ± 1.53	17.52[c] ± 0.89	4, 10	49.17	<0.001
P. aeruginosa	11.14[a] ± 1.11	13.57[ab] ± 0.93	10.32[a] ± 1.38	13.96[b] ± 1.30	17.82[c] ± 1.99	4, 10	13.32	0.001
E. coli	10.07[a] ± 0.76	12.35[ab] ± 0.48	9.83[a] ± 1.50	13.12[b] ± 1.10	15.82[c] ± 0.39	4, 10	20.45	<0.001
S. aureus	11.50[a] ± 1.02	15.02[b] ± 0.39	9.54[a] ± 0.78	17.00[bc] ± 0.35	18.33[c] ± 1.62	4, 10	45.03	<0.001

Supplementary Table S5. Antibacterial activities of HIL 80 % methanol extracts

Test pathogens	Rabbit manure	Potato waste	Pig manure	Market waste	Positive control	DF	F-Value	P-Value
B. subtilis	6.80[a] ± 0.23	8.21[b] ± 0.60	6.87[a] ± 0.12	9.25[c] ± 0.21	16.43[d] ± 1.19	4, 10	588.2	<0.001
P. aeruginosa	6.91[a] ± 0.30	9.58[b] ± 0.38	6.91[a] ± 0.31	9.07[b] ± 0.31	15.90[c] ± 0.43	4, 10	602.1	<0.001
E. coli	6.95[a] ± 0.86	8.52[b] ± 0.47	6.84[a] ± 0.32	9.40[b] ± 0.43	16.37[c] ± 0.60	4, 10	142.6	<0.001
S. aureus	6.87[a] ± 0.07	8.40[b] ± 0.44	7.09[a] ± 0.19	9.34[b] ± 0.20	16.21[c] ± 0.65	4, 10	311.9	<0.001
